# A cross-sectional survey on knowledge and attitudes of Greek dentists regarding molar incisor hypomineralisation diagnosis and treatment

**DOI:** 10.1186/s12903-022-02525-3

**Published:** 2022-11-16

**Authors:** Kyriaki Seremidi, Stefanie Amend, Norbert Krämer, Sotiria Gizani

**Affiliations:** 1 Department of Pediatric Dentistry, Athens School of Dentistry, National & Kapodistrian University of Athens, 2 Thivon str., 11527 Goudi, Athens Greece; 2 Department of Pediatric Dentistry, Medical Center for Dentistry, University Medical Center Giessen and Marburg (Campus Giessen), Justus-Liebig-University Giessen, Schlangenzahl 14, 35392 Giessen, Germany

**Keywords:** Molar incisor hypomineralisation, Knowledge, Attitudes, Awareness, Diagnosis, Treatment

## Abstract

**Background:**

Molar Incisor Hypomineralisation (MIH) remains a challenge for clinicians underlining the gap in the literature regarding the condition. The study aimed to record knowledge and attitudes of Greek dentists regarding diagnosis and treatment of MIH and correlate findings with non-dental characteristics.

**Methods:**

It is a cross-sectional study based on a questionnaire consisting of 37 multiple-choice questions. Data regarding diagnosis, aetiopathogenesis, and clinical management of MIH were collected from active members of three Greek Dental Associations. Chi-square and student’s t-test were used to correlate responses with practitioners’ characteristics and odds ratios calculated to evaluate differences on treatment of MIH-affected teeth (*p* < 0.05).

**Results:**

From the 360 participants (response rate = 94%), 185 were general dental practitioners (GDPs) and 175 dental specialists (59 paediatric dentists (PDs), 38 orthodontists and 78 of other specialties).MIH was commonly encountered as a clinical problem, with GDPs reporting genetics and fluoride intake as common aetiological factors at significantly higher percentages as compared to PDs (*p < 0.05*). Permanent molars and incisors (44%) were the teeth most commonly affected, with yellow/brown demarcated opacities (68%) the most common clinical feature with PDs reporting them in a significantly higher percentage (*p < 0.05)*. Dentists with specialisation, dentists that treat > 10 children per week and children with MIH-affected teeth had a 2–5.5 times greater probability to report difficulty achieving sufficient anaesthesia and hypersensitivity problems (*p < 0.001*). Agreement between GDPs’ and dental specialists’ views was found on less invasive treatment of anterior lesions. Non-PDs reported bulk-fill restorations and onlays as the ideal treatment of severely-affected posterior teeth, as compared to PDs that preferred preformed metal crowns (*p < 0.05*). Multivariate logistic regression analysis revealed that the age of the clinician, years of experience and number of children treated per week were the factors significantly associated with the decision for the treatment of only severely-affected posterior MIH teeth.

**Conclusions:**

Most participants had encountered MIH-affected teeth in their clinical practice and were able to recognize main aetiological factors and clinical findings related to the condition. Nevertheless, their knowledge regarding treatment is limited.

**Supplementary Information:**

The online version contains supplementary material available at 10.1186/s12903-022-02525-3.

## Background

Molar Incisor Hypomineralisation (MIH) is a developmental enamel defect, of systemic origin, affecting at least one first permanent molar and usually associated with lesions in the permanent incisors [1]. With a prevalence that ranges between 8 and 21% globally, and between 4 and 25% in Europe, MIH still remains a contemporary topic for academics and researchers as well as a challenge for the clinician as there are still areas that need to be illuminated [1].

Over the past decades specific guidelines have been developed to improve knowledge regarding diagnosis, clinical features and treatment modalities of MIH-affected teeth. Due to the unsatisfactory data situation on the epidemiology, aetiology and therapy of MIH, the recommendations are partly contradictory, which could influence the dentists’ knowledge differently [1, 2]. At the same time, compliance with the guidelines and their successful implementation is not yet fully known.

Up to date, there is limited evidence, from questionnaire-based surveys in Europe, Asia and Australia [3–12] regarding knowledge and attitudes of dentists about MIH. All studies reported that it is a widespread clinical problem, whose diagnosis and management is challenging. Results showed variety of responses regarding aetiopathogenesis and treatment, underlying that there is a confusion about these parameters mainly among General Dental Practitioners (GDPs).

Despite various studies around Europe having reported on both students and qualified dentists’ knowledge and attitudes on MIH, data yet to be collected in Greece. With a prevalence that ranges between 10 and 21%, in Greece [13, 14], and taking into consideration the fact that a considerable number of children are treated by non-specialized practitioners, investigation of the total knowledge is essential. Therefore, the aim of the study was to record knowledge and attitudes of Greek dentists regarding diagnosis and treatment of MIH. Secondary objective was to correlate the above findings with non-dental factors such as main characteristics of the participants in order to optimize the knowledge of the clinicians about proper diagnosis and treatment of the condition.

## Methods

It is a cross-sectional survey that involved the completion of a computer-based questionnaire completed by dentists, members of the three largest Greek Dental Associations (Athens, Piraeus and Thessaloniki). The study was performed in accordance to the Declaration of Helsinki (WMA 2013) ethical standards and the research protocol was approved by the Ethics Committee of the School of Dentistry, National & Kapodistrian University of Athens, Greece (N473, approved on 08/10/2021). A letter containing the information regarding the aim and the protocol of the study along with the questionnaires was mailed to Dental Associations and acceptance of collaboration was approved by the boards.

### Sample

The sample consisted of active members of the three Associations, with no further restrictions regarding the characteristics of the participants applied.

The sample size was estimated using the equation *n* = n0/Ν, where N is the total number active members in all three associations and n0 = Z^2^_1-a/2_ p (1-p)/e^2^. Given Z^2^_1-a_ has a value of 1.96 as significance level a is equal to 5%, p was equal to 0.5 and e representing the highest accepted error was given a value of 0.05, n0 was calculated equal to 384.5. Since *N* = 7957, n0/N equals to 0.048 and as *n* < 0.05, final sample size ***n*** equals n and was therefore 385.

### Data collection

Data regarding demographic characteristics of the dentists and MIH-related parameters were collected using a computer-based questionnaire (google forms, web application, google, docs.google.com/forms, google LLC, Mountain view, CA, USA). It consisted of 37 multiple-choice questions, divided into seven sections (Additional file 1: Appendix 1). First section focused on sociodemographic data including gender, age, dental qualification and acquisition of post-graduate degrees. Data regarding years of experience and practicing dentistry and mean number of paediatric patients treated weekly were also collected. In the following sections participants were asked to report on their knowledge regarding MIH, dentition and teeth most commonly affected, diagnostic tools and possible aetiological factors. Information on differential diagnosis, common problems patients face and chief complaint seeking dental care were also collected. Questions regarding management of MIH-affected teeth followed focusing on concepts used to decide upon treatment and main problems faced during treatment, treatment options for hypersensitivity and treatment for moderately and severely-affected anterior and posterior teeth. Clinical photos were available to help respondents decide upon answering. Last section of the questionnaire included questions about participants’ need for improving their knowledge and continuing education and clinical training regarding MIH.

Data were collected between November 2021 and March 2022. The survey link was sent by each Dental Association to their members and completed electronically by the participants. Participants agreed upon participation by completing the questionnaire. No personally identifiable data could be obtained by the researchers and the participants maintained their anonymity throughout. Reminder emails were sent to all one and two months after the initial mailing.

Prior to the initiation of the survey, the questionnaire was piloted to test applicability and repeatability amongst 35 dentists, active members of the above dental associations. Results indicated high validity (k = 0.93) and repeatability (k = 0.87) and minor revisions were made on the original version of the questionnaire after the comments of the participants, mainly regarding the quality of the pictures used and syntax and grammatical errors. No major ambiguities were reported for most questions.

### Data analysis

Data from google forms were collected into an Excel spreadsheet (Microsoft Excel, Microsoft Corporation, Redmond, WA, USA) and analysed using SPSS Version 21.0 (SPSS Inc., Chicago, IL, USA). The first part provided the descriptive profile of the sample, which was divided into four major categories according to specialty acquisition (i.e. GDPs, Paediatric Dentists (PDs), orthodontists, dentists practicing other specialties). Distribution of answers to the questions regarding all aspects of MIH were reported according to the above categorization. Variables where multiple responses were allowed, were computed into binary for each specific response and the percentages for a positive response were reported. Significance of calculated differences were tested using Chi-square test and students’ t-test and the statistical significance was set at *p* < 0.05.

Odds ratio (95% confidence intervals) were also calculated according to variables associated to professional experience, to present probability of reporting specific problems during treatment and treatment of hypersensitivity for clinicians that were more familiar with MIH. Multivariate logistic regression analysis without forward and backward elimination of nonsignificant predictors was utilized (deletion criterion using Pearson Chi-square test significance level was at *p* > 0.05), to identify the effect of various non-dental factors upon the decision of treatment of MIH-affected teeth. For the treatment of anterior teeth, minimally invasive treatment options (bleaching, microabrasion, sandblast and infiltration) were set as the dependent variable, with more aggressive schemes (onlays, preformed metal crown and extraction) being the corresponding dependent variable for the treatment of posterior MIH-affected teeth.

## Results

### Sample

From the 385 participants that were recruited for the survey, 360 completed the questionnaire, resulting in a response rate of 94%. The characteristics of the sample are presented in Table 1. Overall, sample consisted of 185 GDPs and 175 dental specialists, of which 59 in paediatric dentistry, 38 in orthodontics, 27 in prosthodontics, 13 in operative dentistry, 10 in endodontics and the remaining 28 in other specialties. Majority of the respondents were females (64%), aged between 41 to 50 years (33%), had graduated from the National and Kapodistrian University of Athens (67%), and had a working experience of ≥11 years (70%). They mainly work in the private sector (95%) and they treat < 5 children per week (42%), with the exception of PDs and orthodontists, vast majority of whom treat > 20 children per week (86 and 84%, respectively).

**Table 1:** Demographic characteristics of the respondents

	GDPs (***N*** = 185)	Paediatric Dentists (***N*** = 59)	Orthodontics (***N*** = 38)	Dentists of other Specialties (***N*** = 78)	Total (***N*** = 360)
	N (%)	N (%)	N (%)	N (%)	N (%)
*Gender*					
Male	68 (37)	7 (12)	18 (47)	35 (45)	128 (36)
Female	117 (63)	52 (88)	20 (53)	43 (55)	232 (64)
*Age*					
< 30 yrs	49 (27)	1 (2)	1 (3)	6 (8)	57 (16)
31–40 yrs	30 (16)	23 (40)	9 (24)	19 (24)	81 (23)
41–50 yrs	47 (25)	25 (42)	12 (32)	34 (44)	118 (33)
51–60 yrs	39 (21)	7 (12)	10 (26)	18 (23)	74 (21)
> 60 yrs	20 (11)	3 (5)	6 (16)	1 (1)	30 (8)
*University*					
NKUA	131 (71)	36 (61)	16 (42)	58 (74)	241 (67)
AUT	11 (6)	13 (22)	11 (29)	7 (9)	42 (12)
Dental School in Europe	43 (23)	8 (14)	11 (29)	13 (17)	75 (21)
Dental School in USA	0 (0)	2 (3)	0 (0)	0 (0)	2 (1)
*Working experience*					
< 5 yrs	52 (28)	2 (3)	1 (3)	6 (8)	61 (17)
5–10 yrs	16 (9)	15 (25)	5 (12)	11 (15)	47 (13)
11–20 yrs	52 (28)	23 (40)	15 (40)	38 (48)	128 (36)
> 20 yrs	65 (35)	19 (32)	17 (45)	23 (29)	124 (34)
*Working place*					
Private sector	178 (52)	56 (17)	37 (11)	73 (21)	342 (95)
Public sector	14 (4)	3 (1)	3 (1)	3 (1)	23 (6)
University	0 (0)	8 (2)	3 (1)	10 (3)	21 (6)
*Treatment of children*					
None	31 (17)	0 (0)	0 (0)	29 (37)	60 (17)
< 5	110 (60)	0 (0)	3 (8)	37 (47)	150 (42)
5–10	22 (12)	1 (2)	0 (0)	10 (13)	33 (9)
11–20	10 (5)	7 (12)	3 (8)	1 (1)	21 (6)
> 20	12 (7)	51 (86)	32 (84)	1 (1)	96 (27)

*GDPs* General Dental Practitioners, *NKUA* National and Kapodistrian University of Athens, *AUT* Aristotle University of Thessaloniki

### Knowledge and diagnosis

**Fig. 1:**
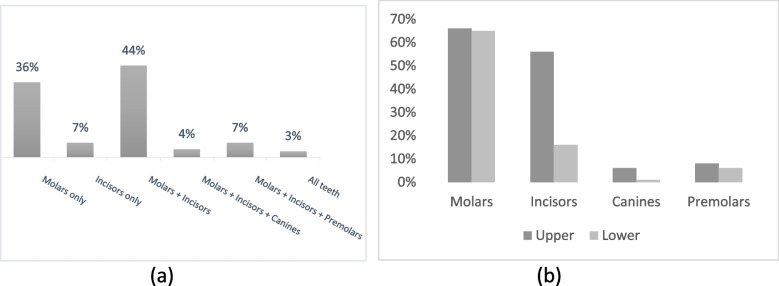
Distribution of most commonly affected teeth by (a) group and (b) type of teeth

Most of the respondents (92%) reported that they know MIH, with 78% reporting undergraduate studies as their main source of knowledge (Table 2). Seminars and continuing education courses were reported significantly more often by PDs (64%) (*p = 0.02*) and orthodontists (50%) (*p = 0.04)* as compared to GDPs (48%) and dentists of other specialties (39%). Majority of the participants reported that MIH is a problem of permanent dentition with 44% reporting that both molars and incisors are affected, 36% only molars and 7% only incisors (Fig. 1), with the differences not being statistically significant.

**Table 2:** Perception of the participants regarding basic knowledge on MIH

	GDPs	Paediatric Dentists	Orthodontics	Dentists of other Specialties	Total
	N (%)	N (%)	N (%)	N (%)	N (%)
*Knowledge*					
Yes	168 (91)	59 (100)	38 (100)	67 (86)	332 (92)
No	17 (9)	0 (0)	0 (0)	11 (14)	28 (8)
*Source of knowledge*					
Undergraduate studies	139 (75)	56 (95)	31 (82)	53 (68)	279 (78)
Seminars/Continuing education*	89 (48)	38 (64)**	19 (50)**	30 (39)	176 (49)
Periodicals	25 (14)	15 (25)	12 (32)	15 (19)	67 (19)
Colleagues	35 (19)	2 (3)	7 (18)	13 (17)	57 (16)
*Dentition*					
Primary	34 (18)	5 (9)	2 (5)	7 (9)	48 (13)
Permanent	151 (82)	54 (92)	36 (95)	71 (91)	312 (87)
*Teeth most commonly affected*					
Molars only	61 (33)	27 (46)	14 (37)	27 (35)	129 (36)
Incisors only	15 (8)	0 (0)	1 (3)	9 (12)	25 (7)
Molars + Incisors	81 (44)	30 (51)	18 (47)	31 (40)	160 (44)
Molars + Incisors + Canines	5 (3)	2 (3)	2 (5)	4 (5)	13 (4)
Molars + Incisors + Premolars	17 (9)	0 (0)	2 (5)	5 (6)	24 (7)
All teeth	6 (3)	0 (0)	1 (3)	2 (3)	9 (3)
*Aetiopathogenesis*					
Genetics*	152 (82)**	39 (66)	30 (79)	65 (83)**	286 (79)
Antibiotics	109 (59)	35 (59)	23 (61)	33 (42)	200 (56)
Chronic medical conditions*	65 (35)	33 (56)**	5 (13)	30 (39)	133 (37)
Acute medical conditions	54 (29)	23 (39)	13 (34)	21 (27)	111 (31)
Fluoride*	56 (30)**	3 (5)	8 (21)	15 (19)	82 (23)
Environmental contaminants*	83 (45)	39 (66)**	18 (47)	29 (37)	169 (47)
Other	30 (16)	14 (24)	3 (8)	14 (18)	61 (17)
*Most common dental problems*					
Pain*	31 (17)	26 (44)**	12 (32)	16 (21)	85 (24)
Hypersensitivity*	97 (52)	51 (86)**	23 (61)	44 (56)	215 (60)
Loss of tooth structure	134 (72)	34 (58)	26 (68)	49 (63)	243 (68)
Aesthetic concerns	140 (76)	38 (64)	26 (68)	52 (67)	256 (71)

*statistical significance *p* < 0.05 with Chi-square test

**statistical significance *p* < 0.05 with Student’s t-test

*GDPs* General Dental Practitioners

A variety of views were expressed regarding aetiopathogenesis, with more than half of the respondents reporting a combination of factors. Almost 80% reported that there is a genetic component, 56% that administration of medications is involved and almost half (47%) that there is an external influence by environmental contaminants. Significant differences were recorded with GDPs (*p = 0.04*) and dentists of other specialties (*p = 0.03*) reporting genetics and fluoride intake at higher percentages as compared to PDs and orthodontists. PDs reported chronic medical conditions and environmental contaminants at significantly higher percentages as compared to both GDP and dentists of any other specialty (*p = 0.02*). Although, aesthetics and tooth structure loss were the two most commonly reported problems in percentages > 65, with pain and hypersensitivity being reported significantly more often by PDs as compared to GDPs and other specialties (*p = 0.01*).

Regarding diagnosis (Table 3), almost all participants base their diagnosis on clinical examination, with 11% adding radiographic examination and 3% reporting no use of any diagnostic tool. Yellow/brown opacities (68%) were the most commonly reported clinical feature, with PDs (*p = 0.01*) and orthodontists (*p = 0.02*) reporting them in a significantly higher percentage as compared to GDPs and dentists of other specialties. From the conditions resembling MIH, white spot lesions were correctly identified by more than 2/3 of the participants (82%) and fluorosis by more than 1/3 (37%). Amelogenesis imperfecta was correctly identified by more than half of all specialists with GDPs reporting significantly lower percentages (*p = 0.01*).

**Table 3:** Responses regarding diagnosis of MIH

	GDPs	Paediatric Dentists	Orthodontics	Dentists of other Specialties	Total
	N (%)	N (%)	N (%)	N (%)	N (%)
*Diagnosis based on*					
Clinical examination	176 (95)	59 (100)	35 (92)	70 (90)	340 (94)
Radiographs	18 (10)	9 (15)	5 (13)	7 (9)	39 (11)
Fluorescence devices	13 (7)	1 (2)	2 (5)	8 (10)	24 (7)
None	7 (4)	0 (0)	1 (3)	4 (5)	12 (3)
*Main clinical characteristics*					
White-creamy lesions	113 (61)	38 (64)	18 (47)	41 (53)	212 (59)
Brown-yellow lesions*	115 (62)	48 (81)**	28 (74)**	54 (69)	245 (68)
Atypical restorations	69 (37)	25 (42)	14 (37)	20 (26)	128 (36)
Tooth structure loss	64 (35)	29 (49)	11 (29)	26 (33)	130 (36)
*Differential Diagnosis from conditions that resemble MIH*					
Fluorosis	67 (36)	29 (49)	12 (32)	24 (31)	132 (37)
Amelogenesis Imperfecta*	85 (46)**	40 (68)	25 (66)	43 (55)	193 (54)
White spot lesions	144 (78)	53 (90)	31 (82)	68 (87)	296 (82)

*GDPs* General Dental Practitioners

*statistical significance *p* < 0.05

**statistical significance *p* < 0.05 with Student’s t-test

### Treatment

Regarding treatment vast majority of the participants reported that they base their decision upon clinical examination (80%), severity of the lesion (91%) and caries risk of the patient (74%), with no statistically significant differences in the reported values. Significant differences were only calculated for the guidelines by paediatric associations with almost ¾ of PDs supporting their use, as compared mainly to GDPs (38%).

Various clinical challenges posed by MIH were reported, with 70% of the respondents mentioning difficulties with adhesion as their main problem during treatment. PDs, dentists that treat > 10 children per week and children with MIH-affected teeth had a 2–5.5 times greater probability to report difficulty achieving sufficient anaesthesia and hypersensitivity problems in these patients (Table 4). Similarly, dentists that treat > 10 children and children with MIH-affected teeth are 2–4.5 more likely to report difficulties in patients’ co-operation.

**Table 4:** Odds ratio (confidence interval) for reporting specific problems during treatment, according to clinicians’ clinical experience

	***Sufficient Anaesthesia***^a^ ***(31%)***			***Cavity Design***^a^ ***(42%)***			***Adhesion***^a^ ***(70%)***			***Hypersensitivity***^a^ ***(39%)***			***Co-operation***^a^ ***(25%)***			***None***^a^ ***(9%)***		
	***OR***	***95% CI***	p-value**	***OR***	***95% CI***	p- value **	***OR***	***95% CI***	p- value **	***OR***	***95% CI***	p- value **	***OR***	***95% CI***	p- value **	***OR***	***95% CI***	P -value*
**Years of experience**																		
≤ 10 yrs	1.7	0.9-	**0.05**	0.7	0.4-	0.25	1.9	1.1-	**0.03**	1.7	1–2.8	**0.04**	1.5	0.9-	0.15	0.93	0.4-	0.9
> 10 yrs	Ref	2.9		Ref	1.2		Ref	3.5		Ref			Ref	2.6		Ref	2.3	
**Specialisation**																		
Yes	2.8	1.65-	**< 0.01**	0.7	0.46-	0.16	1.5	0.89-	0.14	1.6	1-	**0.04**	1.5	0.87-	0.15	0.5	0.23-	0.13
No	Ref	4.65		Ref	1.1		Ref.	2.38		Ref	2.56		Ref	2.48		Ref	1.2	
**Children treated**																		
> 10	5.5	3.2-	**< 0.01**	0.6	0.37-	**0.03**	1.1	0.7-	0.6	3.1	1.9-	**< 0.01**	4.5	2.6-	**< 0.01**	0.5	0.2-	0.12
≤ 10	Ref	9.5		Ref	0.95		Ref	1.9		Ref	4.9		Ref	17.8		Ref	1.2	
**Children with MIH-affected teeth**																		
Yes	4.7	2.1-	**< 0.01**	1.5	0.9-	0.17	1.1	0.6-	0.8	2.6	1.4-	**0.02**	2	1–4	**0.04**	0.4	0.2-	**0.01**
No	Ref	10.2		Ref	2.5		Ref	1.9		Ref	4.7		Ref			Ref	0.8	

*OR* Odds Ratio, *CI* Confidence Interval

^a^Reference unit: negative control

**Pearson Chi-square test was used for all variables and bold values indicate statistical significance (*p* < 0.05)

Microabrasion (34%) and no treatment (37%) were the choices of most participants for the treatment of anterior lesions (Table 5). Twenty seven percent of PDs reported resin infiltration and 24% of GDPs composite resin as a possible treatment, with the differences not being statistically significant. Respectively, preformed metal crowns (PMC) (38%) and composite resin (29%) were the most preferred treatment for moderate/severe posterior lesions (Table 5). Significant differences existed between PDs that reported PMC (*p = 0.01*) in a vast majority (70%) and in almost double percentages compared to GDP (34%) and other specialties (28%). GDPs reported the use of bulk-fill restorations in a significantly higher percentage as compared to dentists of any specialty (*p = 0.04*).

**Table 5:**
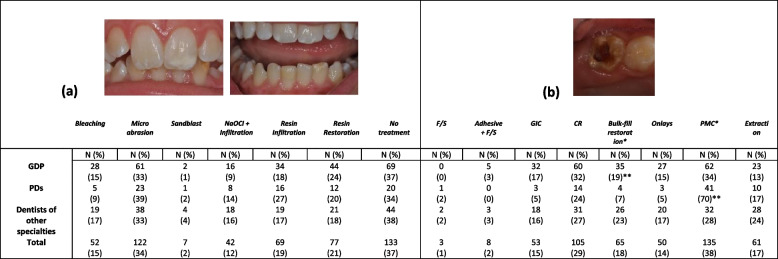
Distribution of responses regarding treatment of MIH-affected teeth: (a) mild anterior lesions and (b) moderate/severe posterior lesions

*GDPs* General Dental Practitioners, *PDs* Paediatric Dentists, *F/S* Fissure Sealing, *GIC* Glass-ionomer Cement, *CR* Composite Resin, *PMC* Preformed Metal Crown

*indicating statistically significant differences

**statistical significance *p* < 0.05 with student’s t-test

Multivariate logistic regression analysis (Table 6) revealed that age of the clinician, years of experience and number of children treated per week were the factors significantly associated with the decision for the treatment of only severely-affected posterior teeth. Practitioners aged > 40 years, with more years of experience and those treating more children per week have a 2–4 greater probability to choose more aggressive treatments as compared to those with less years of experience and those that do not treat patients with MIH. Decision of treatment of mildly-affected anterior teeth is not significantly affected by any of the factors associated with the experience of the practitioners.

**Table 6:** Multivariate logistic regression analysis for the factors affecting decision upon treatment of MIH-affected teeth

	***Anterior Lesion***^a^			***Posterior Lesion***^b^		
	***OR***	***95% CI***	***p-value†***	***OR***	***95% CI***	***p-value†***
**Gender**						
Male	1	0.35–2.79	0.99	1.19	0.72–1.98	0.50
Female	Ref			Ref		
**Age**						
> 40 yrs	0.58	0.18–1.84	0.36	2.28	1.20–4.34	**0.01**
Up to 40 yrs	Ref			Ref		
**Specialisation**						
Yes	0.91	0.31–2.66	0.91	1.37	0.80–2.35	0.26
No	Ref			Ref		
**Years of experience**						
Up to 10	0.46	0.17–1.23	0.46	3.77	2.05–6.93	**< 0.01**
> 10	Ref			Ref		
**Children treated**						
> 10	0.80	0.24–2.68	0.80	2.23	1.23–4.04	**0.01**
≤ 10	Ref			Ref		
**Children with MIH-affected teeth**						
Yes	1.37	0.40–4.66	0.99	0.70	0.38–1.29	0.26
No	Ref			Ref		

*OR* Odds Ratio, *CI* Confidence Interval

^a^Reference unit: more aggressive treatment

^b^Reference unit: less aggressive treatment

†Pearson Chi-square test was used for all variables and bold values indicate statistical significance (*p* < 0.05)

Regarding treatment of hypersensitivity (Table 7) practitioners with fewer years of experience, dentists with specialisation and dentists that treat > 10 children per week had an almost 2 times greater probability to recommend additional aids, such as Casein phosphopeptide-Amorphous calcium phosphate (CPP-ACP), to plain toothbrushing with fluoridated toothpaste. Also, dentists that treat > 10 children per week are 1.3 times more likely to recommend use of toothpastes with desensitizing agents as compared to plain fluoridated toothpaste. For treatment of hypersensitivity at the dental office, dentists that treat > 10 children and children with MIH-affected teeth had a greater probability to prefer conservative treatment involving topical fluoride application, fissure sealant and flowable composite resin placement as compared to restorative intervention.

**Table 7:** Odds ratio (confidence intervals) for the choice of different treatment options for hypersensitivity

	Home (Toothbrushing^a^ Vs Additional means)			Home (Fluoridated^a^ Vs Desensitizing agents)			Office (Prevention^a^ Vs Intervention)		
	***OR***	***95% CI***	p-value **	***OR***	***95% CI***	p-value **	***OR***	***95% CI***	p-value **
**Years of experience**									
Up to 10 yrs	1.8	1.1–3.1	**0.03**	1	0.6–1.6	0.95	1	0.6–1.6	0.94
> 10 yrs	Ref			Ref			Ref		
**Specialisation**									
Yes	1.9	1.1–3.2	**0.02**	1.3	0.83–2.1	0.23	0.9	0.54–1.4	0.51
No	Ref			Ref			Ref		
**Children treated**									
> 10	1.8	1.1–3	**0.02**	1.7	1.1–2.8	**0.02**	0.5	0.3–0.8	**0.01**
≤ 10	Ref			Ref			Ref		
**Children with MIH-affected teeth**									
Yes	0.8	0.4–1.3	0.32	1.3	0.8–2.2	0.36	0.6	0.4–1.1	0.09
No	Ref			Ref			Ref		

*OR* Odds Ratio, *CI* Confidence Interval

^a^Reference unit

** Pearson Chi-square test was used for all variables and bold values indicate statistical significance (*p* < 0.05)

Most participants reported a frequency of recalls every 6 months (45%) and 20% that they see the patients whenever necessary. PDs reported a significantly higher percentage for a frequency of 3 monthly recall appointments (50%), as compared to GDPs (17%) and all other specialties (19%). Regarding the main findings during recall appointments, secondary caries and tooth surface loss were mostly reported in comparable percentages (46 and 42% respectively). One third of the respondents reported also failed restorations and hypersensitivity. PDs reported significantly higher percentages for tooth structure loss (62%) and orthodontists and dentists of other specialties for hypersensitivity (46 and 42%) and for failed restorations (50 and 45%) (data not shown).

### Future Proposals

**Fig. 2:**
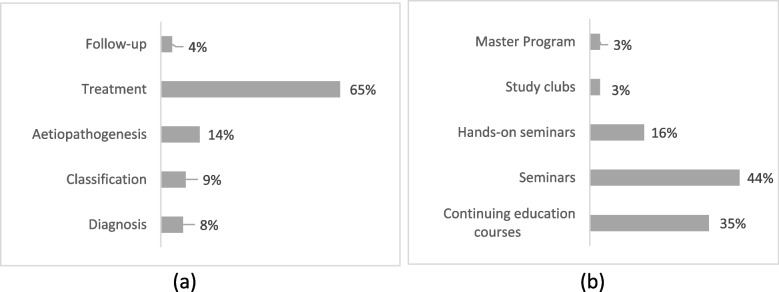
Distribution of responses regarding (**a**) domains and (**b**) ways of improvement

Vast majority of the respondents reported a great need for improving their knowledge regarding treatment of MIH-affected teeth (65%), with the differences amongst specialties not being statistically significant (Fig. 2a). All other domains of improvement were reported in percentages < 15%, indicating a confidence of the respondents regarding diagnosis and aetiopathogenesis. Regarding ways of improvement (Fig. 2b), respondents showed a preference towards seminars (44%) and hands-on courses (35%), with no significant differences in the answers reported.

## Discussion

The current study is the first attempt to explore knowledge and attitudes of Greek dentists regarding MIH, in order to be used as a guide for the organization of continuous educational courses for GDPs and the application of patient-oriented oral healthcare policies. Results showed that Greek dentists in their overwhelming majority (92%) are aware of MIH, with no significant differences between GDPs and specialized dentists. Previous studies have also reported that practitioners in most countries have incorporated MIH in their clinical practice, in percentages that ranged between 65 and 95% [11, 12, 15, 16]. The differences recorded could be attributed to the fact that MIH prevalence varies between different countries and therefore not all practitioners are familiar with the condition. High were also the percentages reported by dental students (95–99%) in most studies [3–5] but for one [17] in which 43% of 4th year and 28% of 5th year dental students in Saudi Arabia have heard of the condition. The difference can be due to the different years the surveys were conducted. It is evident that as years pass the percentage of awareness increases, underlying the widespread nature of the condition that becomes more evident to both students and clinicians.

University lectures were the most commonly reported source of knowledge in the literature. In Greece, dental developmental defects are incorporated in the undergraduate curriculum, educating students how to diagnose MIH, identify its main clinical characteristics and differentiate it from other conditions that resemble in their clinical appearance. PDs reported seminars and continuing education courses as a second source of knowledge in a significantly higher percentage as compared to GDPs. There is an increased interest for Greek specialized dentists towards continuing education through seminars and courses underlying their interest to continuously develop and follow advances in evidence based clinical practice. Such differences have been previously recorded by other authors [11, 18] but not at a statistically significant level.

Almost all respondents (87%) recognized that MIH mainly affects permanent dentition with majority reporting that both molars and incisors are affected. This is confirmed by recent epidemiological data in Greece reporting that 65% of children with MIH have both their molars and incisors affected [19]. The only studies [3, 4] reporting on affected dentition and teeth, showed that dental students in Germany and Austria have encountered lesions also in premolars (65%) and canines (15%), which was the case for only 3 and 9% in our study respectively. One of the contributing factors for the reported differences could be the fact that mainly complicated cases are referred to the dental institutes.

Yellow/brown opacities were the most commonly reported clinical feature in the current study, with PDs identifying them in a significantly higher frequency. This is in accordance with previous studies, reporting that demarcated opacities were perceived by the participants as the most frequently observed lesions [11, 20, 21]. In the current survey, more than one third of the respondents (36%) reported tooth structure loss and atypical restorations as clinical feature, with the differences between GDPs and PDs not being significant. Post-eruptive breakdown has been previously reported in lower percentages [16, 21], as these lesions can be masked by caries and atypical restorations. Combining the above findings regarding MIH diagnosis one could conclude that Greek dentists have a great awareness, with small significant differences occurring between specialized dentists and GDPs.

Despite the established criteria on MIH diagnosis, 42% of Greek dentists misdiagnosed the condition, with white spot lesions being the condition most easily differentiated, while fluorosis the least. This is mainly attributed to the low prevalence of fluorosis in Greece, making difficult for clinicians to be familiar with recognising the clinical features of this particular condition. Difficulty in MIH differential diagnosis has been previously discussed in the literature, and is particularly confounded by amelogenesis imperfecta, fluorosis and early carious lesions [22]. Specialized dentists, such as PDs and orthodontists, more often diagnose MIH correctly. In a previous study less than 1/3 of French orthodontists wrongly diagnosed MIH, compared to 48% of GDPs who did so [9]. This is in agreement with our study, reporting a significantly higher incidence of correctly identifying a case of amelogenesis imperfecta by PDs and orthodontists than GDPs (68 and 66% respectively Vs 46%). This difference could be attributed to the fact that dental defects concern one of the main topics in the post-graduate education of the dental specialties mentioned above and their regular encounternment of these young patients. Moreover, these specialties treat patients during childhood and adolescence more often that GDPs, period during which MIH is easier diagnosed due to dental tissue preservation [23].

A variation in views was recorded about MIH specific aetiological factors, with genetics being the most prevalent factor reported among all respondents, as has been previously pointed out [3, 4, 10]. In the present study, significantly more PDs chose environmental contaminants as a common aetiological factor than GDPs and other specialties (66% Vs 45%), indicating the first ones have a more updated knowledge.

The current survey identified adhesion and cavity design as common barriers clinicians face during management of MIH-affected teeth. PDs showed higher probability for reporting achievement of adequate anaesthesia and problems due to hypersensitivity. These results are not in agreement with findings from most of the studies [7, 18, 22] that mainly report child’s behaviour as the main barrier to care. Multiple dental visits, regular follow-ups and extensive dental interventions increase dental anxiety and have a negative influence on the children’s behaviour. In Greece, behavioural management is achieved using mainly non-pharmacological techniques due to legislation restrictions. This situation results in a thorough training of Greek dentists to apply these techniques in their daily practice as a treatment choice of necessity than in other countries where alternative options (e.g. sedation, general anaesthesia) are also available and easily accessible.

Management of hypersensitivity seems to be of major importance, according to our respondents who advised their patients to use toothpastes with desensitizing agents and additional products for its treatment at home. At practice level, they prefer to apply more often preventive measures such as fluoride and fissure sealants compared to preventive composite resin restorations. Unfortunately, no comparison can be made with the literature since this is the first study, up to our knowledge, reporting data about different approaches for the management of hypersensitivity in MIH-affected teeth.

Composite resin and PMCs were the materials of choice for the treatment of severely-affected molars. These findings are in agreement with some of the previous studies [5, 6, 12, 20, 24] but not in consistent with others [10, 18, 21] that reported glass-ionomer cements (GICs) or resin-modified glass-ionomer cements (RMGICs) as the material of choice. The present study showed that more PDs reported PMCs as the ideal treatment, as has been previously reported [12, 18]. They are more easily applicable by PDs that treat more often severely-affected teeth for which they tend to choose materials with increased longevity. The great variation in treatment protocols among dentists in different countries may indicate a need for more specific guidelines to minimize the treatment burden and secure high quality treatment decisions.

Treatment of anterior lesions included no treatment or microabrasion, with no significant differences identified between respondents. More aggressive intervention has been previously reported [11] with half of GDPs in Kuwait restoring hypomineralised areas with direct composite resin and half of specialized dentists restoring only large lesions. Serna-Munoz et al. reported that GDPs suggest composite resin for the restoration of anterior lesions as compared to PDs that preferred RMGICs with the differences being statistically significant, underlying the more conservative attitudes of the latter [10].

Findings from the current survey highlighted that most participants had encountered MIH in their clinical practice and were able to recognize main aetiological factors and clinical findings related to the condition. Their knowledge regarding treatment of MIH-affected teeth is limited, underlining the lack of evidence from clinical studies in order to clearly define the grey areas of the field [1, 25–27]. There is need to deepen our knowledge on the adhesion of different materials, the nature and the management of the pain and hypersensitivity as well as the long term evaluation of the different types of treatment provided. The severity of the lesion, patients’ level of co-operation and acceptance should be also evaluated and in relation to the parameters mentioned above.

At the same time, seminars and hands-on courses are required to enrich clinicians’ ability to offer appropriate treatment based on patients’ needs and expectations. This has been previously highlighted and underlines the need for a universal standardized protocol for data collection and analysis [23]. In this way, an easily accessible clinical guide using modern educational tools and software can be developed which will be able to provide an evidence-based approach for the management of MIH clinical entity.

### Strengths and limitations

The major strength of the survey was its nationwide design and its high response rate. Also, the sample size was calculated and selected from the three societies of the two biggest cities in Greece, with specialized and non-specialized practitioners being randomly and equally distributed. This allowed direct comparisons to be performed, generalization of the results and specific conclusions to be drawn for Greek dental practitioners.

Although, results should be interpreted with caution due to specific limitations of the survey, such as the over- or under-reporting of the participants. Multiple responses in specific questions further increase response bias as participants can choose from one to all answers that in cases may differ from the decisions they make in their everyday practice. Also, reporting bias could have been produced by the exclusion of participants from other dental societies that are in the suburb. Dentists from these societies are often of older age and have less access to continuing education advanced courses. In addition, a big percentage of the specialized dentists practice in big cities, fact that can further influence the reporting bias.

Finally, despite the variety of questions included to cover major issues regarding MIH, an overall score corresponding to the level of knowledge and confidence could not be obtained and each question was evaluated separately to draw conclusions.

## Conclusions

- MIH was encountered as a clinical problem mainly attributed to genetics, antibiotics and environmental contaminants.
- Permanent molars and incisors were reported as the most commonly affected teeth with yellow/brown demarcated opacities the most prominent clinical presentation.
- Achieving sufficient anaesthesia and hypersensitivity were the most frequently reported barriers.
- Non-invasive treatment was the choice for treatment of anterior lesions and bulk-fill restorations and PMCs for severely-affected posterior teeth.
- Fluoridated toothpaste and desensitizing agents were prescribed for the treatment of hypersensitivity at home while fluoride and fissure sealants for office-use.
- Among Greek dentists knowledge regarding treatment of MIH-affected teeth is limited and therefore there is a great need for continuing education courses to help clinicians provide high quality dental care.

## Supplementary Information

**Additional file 1:.** Appendix 1.

## Data Availability

The datasets used and/or analysed during the current study are available from the corresponding author on reasonable request.

## Abbreviations

MIH: Molar Incisor Hypomineralisation
GDPs: General Dental Practitioners
PDs: Paediatric Dentists
PMCs: Preformed Metal Crowns
CPP-ACP: Casein phosphopeptide-Amorphous Calcium Phosphate
GICs: Glass-Ionomer Cements
RMGICs: Resin-modified Glass-Ionomer Cements
