# Supplementary material for: A cross-sectional survey on knowledge and attitudes of Greek dentists regarding molar incisor hypomineralisation diagnosis and treatment
Source: BMC Oral Health. 2022 Nov 16;22:498. doi: 10.1186/s12903-022-02525-3 (PMC9670472; doi:10.1186/s12903-022-02525-3)
Supplement: Supplementary file 1 — Additional file 1:. Appendix 1. [file 12903_2022_2525_MOESM1_ESM.docx]

NATIONAL AND KAPODISTRIAN UNIVERSITY OF ATHENS

SCHOOL OF DENTISTRY

DEPARTMENT OF PEDIATRIC DENTISTRY

In collaboration with the

Department of Pediatric Dentistry, [Justus-Liebig-Universität Gießen](https://www.researchgate.net/institution/Justus-Liebig-Universitaet-Giessen), Gießen, Hesse, Germany

***KNOWLEDGE AND ATTITUDES OF GREEK DENTISTS REGARDING DIAGNOSIS AND TREATMENT OF MIH TEETH.***

**QUESTIONNAIRE**

**Number**

ΟΟΟ

| Date: __________________________________________________________________ | | | |
| --- | --- | --- | --- |
| Dental Association: |  |  |  |

| ***A*** | ***GENERAL INFORMATIONS*** |  |  |
| --- | --- | --- | --- |
| 1. | In which age group do you belong? | 1. <30 yrs 2. 31-40 yrs 3. 41-50 yrs 4. 51-60 yrs 5. >60 yrs |  |
| 2. | What is your gender? | 1. Male 2. Female |  |
| 3. | Which University have you graduated from? | 1. National and Kapodistrian University of Athens 2. Aristotle University of Thessaloniki 3. University in European Countries 4. University in the USA |  |
| 4. | How many years have you been practicing? | 1. < 5 years 2. 5-10 years 3. 10-20 years 4. > 20 years |  |
| 5. | Do you have a specialization? | 1. Yes 2. No |  |
| 6. | If yes, in which subject | 1. Endodontics 2. Maxillofacial Surgery 3. Oral Diagnosis and Radiology 4. Oral Medicine 5. Oral Surgery 6. Orthodontics 7. Pediatric Dentistry 8. Periodontology 9. Prosthodontics 10. Restorative Dentistry |  |
| 7. | Where do you practice? | 1. Private Sector 2. Public Sector 3. University Setting |  |
| 8. | Do you treat children at your everyday practice? | 1. Yes 2. No |  |
| 9. | If yes, how many in average per week | 1. <5 2. 5-10 3. 11-20 4. >21 |  |
| ***B*** | ***DISEASE RELATED QUESTIONS*** |  |  |
| 10. | Do you know what MIH stands for? | 1. Yes 2. No |  |
| 11. | If yes, what is your source of information? | 1. University 2. Seminars/ Conferences 3. Journals 4. Colleagues/ dental groups on social media 5. Continuing education courses |  |
| 12. | How many patients with MIH (average number per month) do you see in your everyday practice? | 1. None 2. Up to 5 3. 6-10 4. >10 |  |
| 13. | In which dentition do you see it more often? | 1. Primary 2. Permanent |  |
| 14. | Which group of teeth are mainly affected? | 1. Upper molars 2. Upper premolars 3. Upper canines 4. Upper incisors 5. Lower molars 6. Lower premolars 7. Lower canines 8. Lower incisors |  |
| ***C*** | ***DIAGNOSIS RELATED QUESTIONS*** |  |  |
| 15. | Which of the main features of MIH do you recognize in the case? | 1. Lesions in posterior teeth 2. Lesions in anterior teeth 3. White demarcated opacities 4. Yellow/brown demarcated opacities 5. Post eruptive enamel breakdown 6. Atypical restorations | 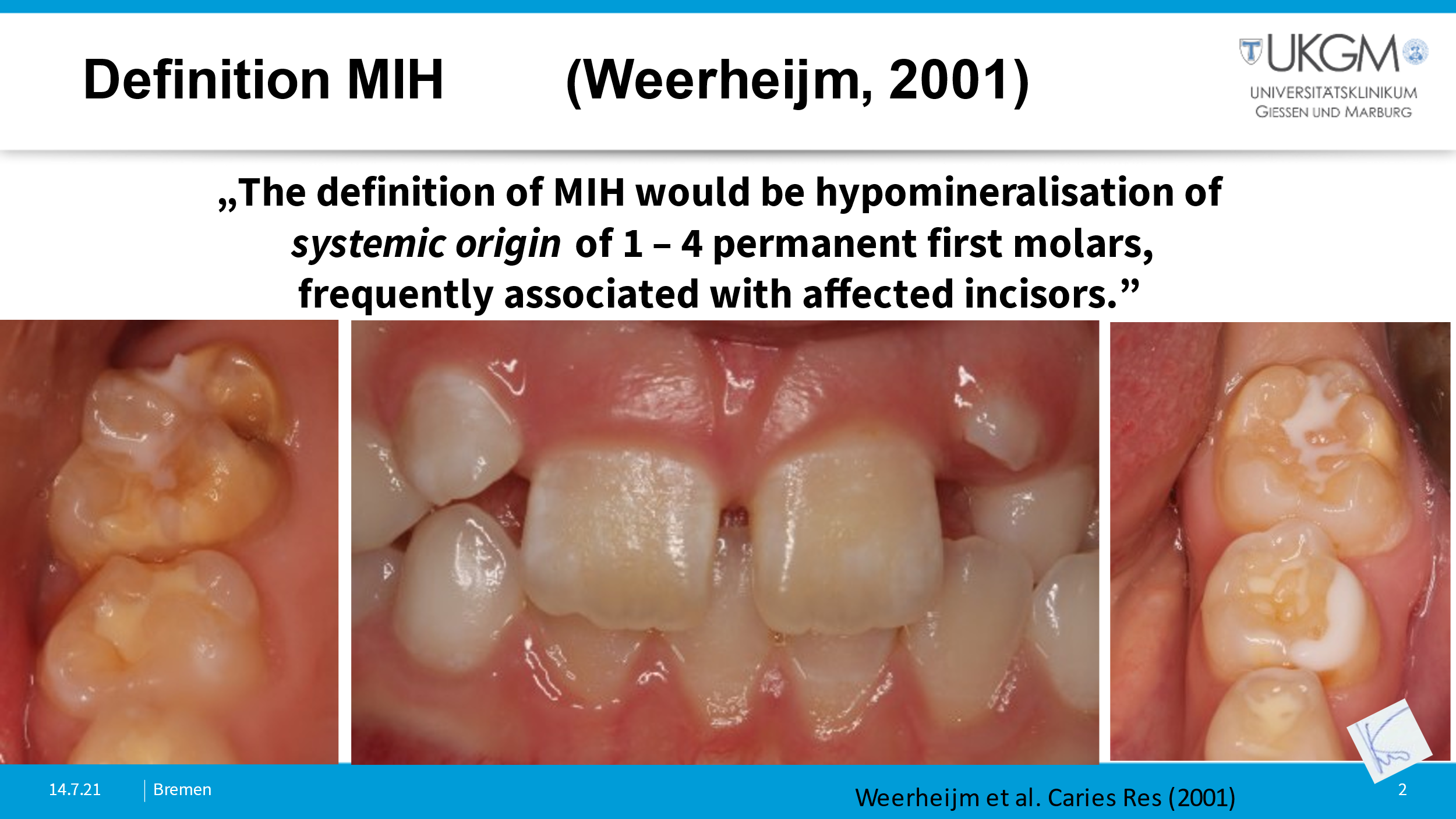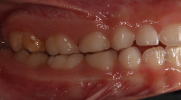 |
| 16. | Which of the main features of MIH do you recognize in the case? | 1. Lesions in posterior teeth 2. Lesions in anterior teeth 3. White demarcated opacities 4. Yellow/brown demarcated opacities 5. Post eruptive enamel breakdown 6. Atypical restorations | 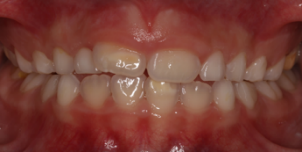  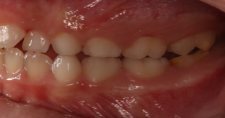 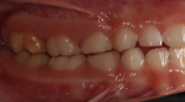 |
| 17. | Which other conditions that resemble MIH do you recognize in the picture? | 1. Amelogenesis Imperfecta 2. Enamel Hypoplasia 3. White spot lesions 4. Fluorosis | 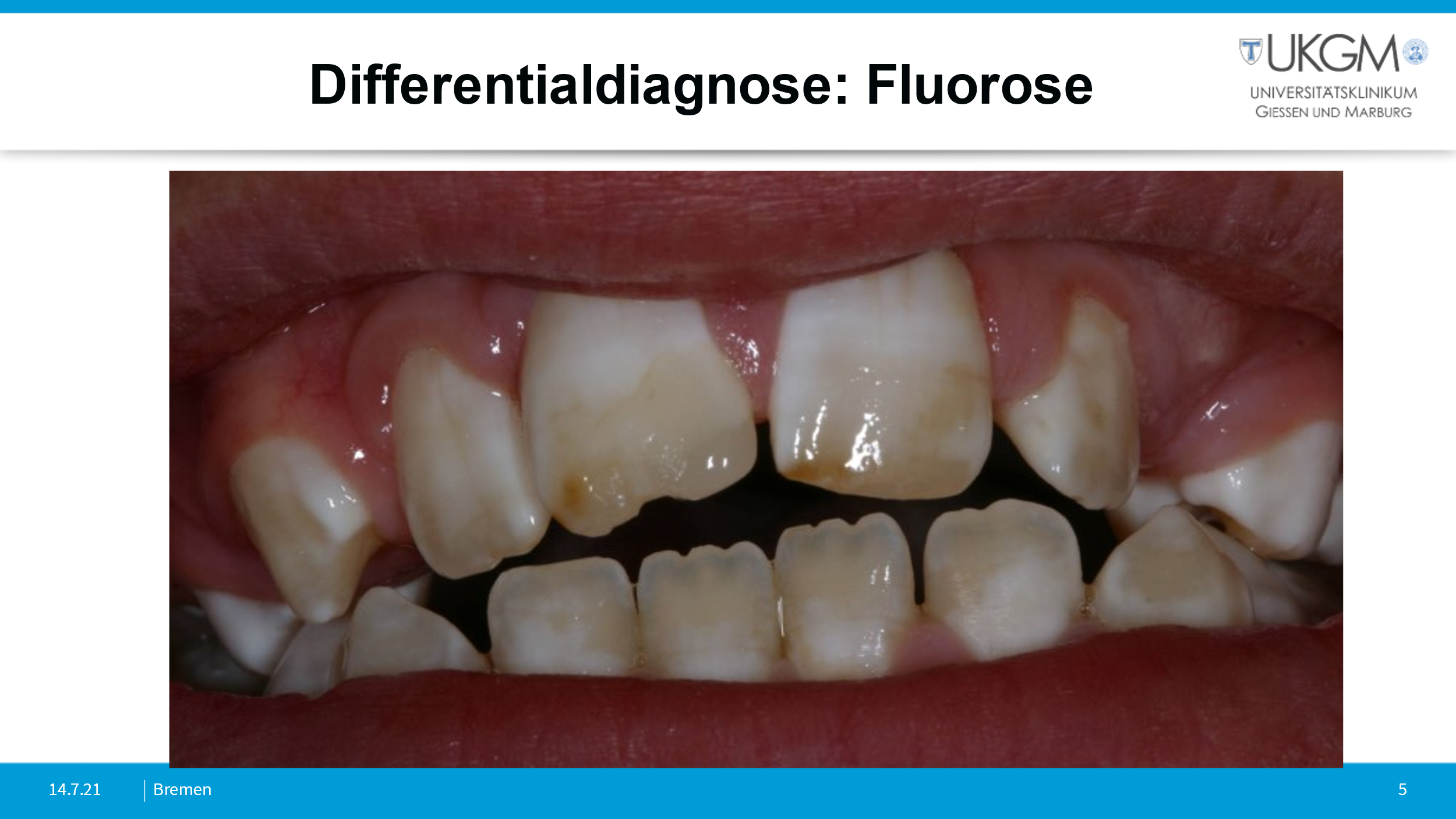 |
| 18. | Which other conditions that resemble MIH do you recognize in the picture? | 1. Amelogenesis Imperfecta 2. Enamel Hypoplasia 3. White spot lesions 4. Fluorosis | 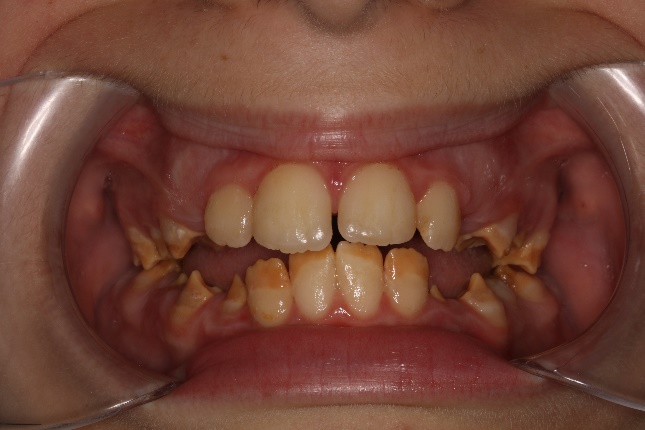 |
| 19. | Which other conditions that resemble MIH do you recognize in the picture? | 1. Amelogenesis Imperfecta 2. Enamel Hypoplasia 3. White spot lesions 4. Fluorosis | 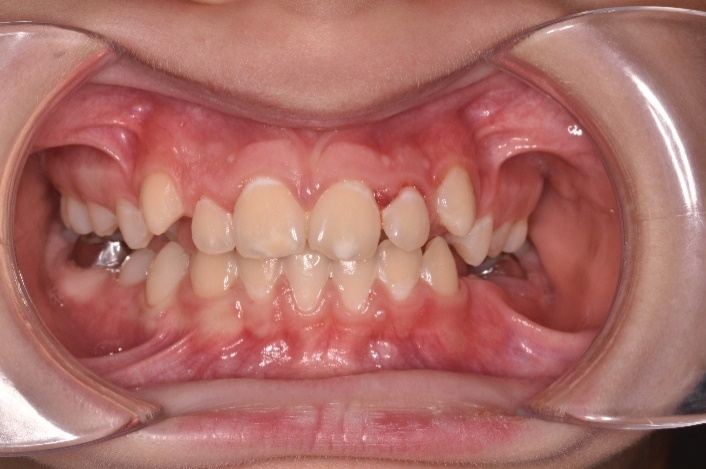 |
| 20. | Do you use a specific diagnostic tool to register the lesions? | 1. Plain registration through standard clinical examination 2. Fluorescence devices (e.g. DIAGNOdent, FOTI, etc) 3. Radiographs 4. None |  |
| 21. | What is the chief complaint of these patients seeking dental care? | 1. Pain 2. Sensitivity to cold 3. Sensitivity during tooth-brushing 4. Enamel chipping 5. Aesthetics |  |
| 22. | Do you assess the influence of MIH on patient’s QoL | 1. Yes 2. No |  |
| 23. | Which domain is mainly affected? | 1. Functional well-being 2. Socio-emotional well-being 3. School activities 4. Self perception |  |
| ***D*** | ***AETIOPATHOGENESIS RELATED QUESTIONS*** |  |  |
| 24. | Which of the following do you think are considered as possible aetiological factors? | 1. Genetics 2. Antibiotics 3. Chronic medical conditions 4. Acute medical conditions 5. Fluoride 6. Environmental contaminants 7. Others |  |
| ***E*** | ***MANAGEMENT RELATED QUESTIONS*** |  |  |
| 25. | Which concept do you use to decide upon treatment | 1. Pure clinical evaluation 2. Caries risk assessment 3. Official guidelines (EAPD/AAPD) 4. Würzburg TNI 5. Patient’s preference 6. Others |  |
| 26. | Does severity of MIH and extent of the lesion affect your decision? | 1. Yes 2. No 3. Don’t know |  |
| 27. | What are the main problems you face during treatment? | 1. Anaesthesia 2. Cavity design and tissue removal 3. Adhesion 4. Margins of restoration 5. Hypersensitivity 6. Difficulties in cooperation 7. None |  |
| 28. | How much hard tissue do you remove during cavity preparation? | 1. Entire hypomineralized/defective enamel 2. Both hypomineralized enamel and unsupported enamel 3. Removal of the porous enamel until resistance to the bur or to the probe is felt 4. Don’t know |  |
| 29. | Which means for home use would you suggest for the treatment of hypersensitivity? | 1. Brushing with fluoridated tooth paste 2. Use of desensitizing tooth paste 3. Use 8% Arginine containing toothpaste 4. Use of CPP-ACP 5. Don’t know |  |
| 30. | How do you treat hypersensitivity of MIH-affected teeth in your practice? | 1. Topical fluoride application 2. Fissure seal 3. Placement of restoration 4. Placement of stainless steel crowns 5. Extraction 6. Don’t know |  |
| 31. | Which of the following you would consider appropriate for the treatment of a moderately affected molar? | 1. Fissure sealant 2. Adhesive + Fissure Sealant or Flowable 3. Glass ionomer cement 4. Resin restoration 5. Bulk Fill restorations 6. Onlays 7. Preformed crowns 8. Extraction | 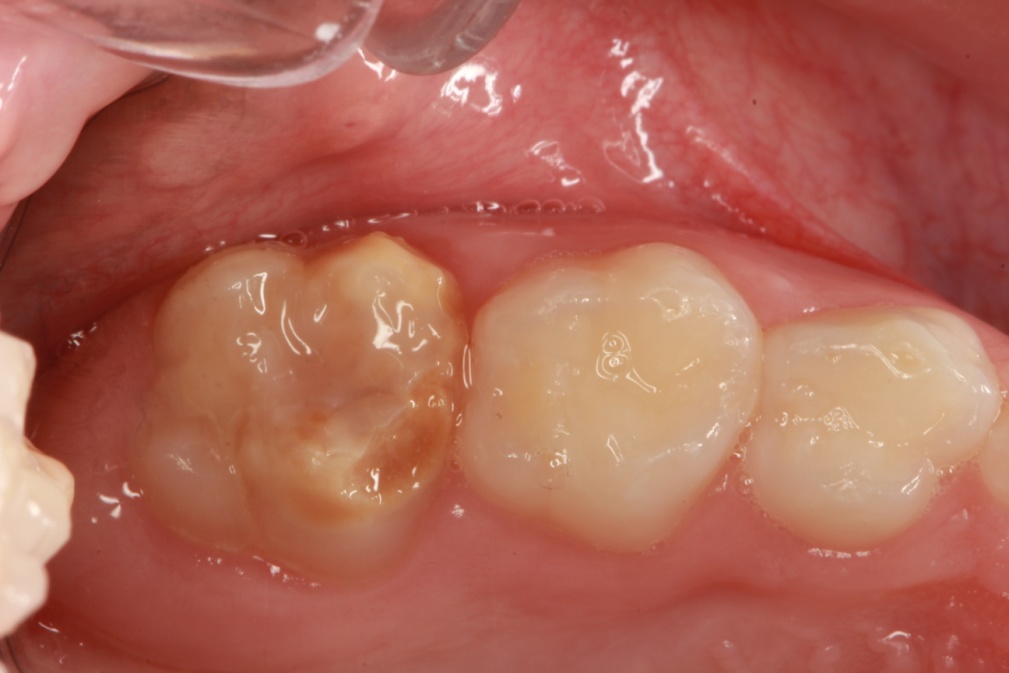 |
| 32. | Which of the following you would consider appropriate for the treatment of a severely affected molar? | 1. Fissure sealant 2. Adhesive + Fissure Sealant/ Flowable 3. Glass ionomer cement 4. Resin restoration 5. Bulk Fill restorations 6. Onlays 7. Preformed crowns 8. Extraction | 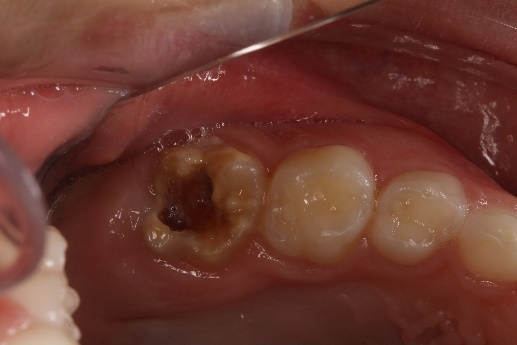 |
| 33. | Which of the following you would consider appropriate for the treatment of a severely affected incisor? | 1. Bleaching 2. Microabrasion 3. Sandblasting 4. NAOCL+ Resin Inflitration 5. Resin infiltration 6. Resin Restoration 7. No treatment | 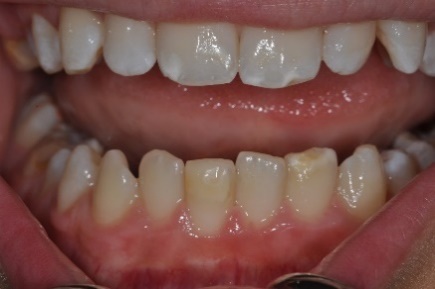  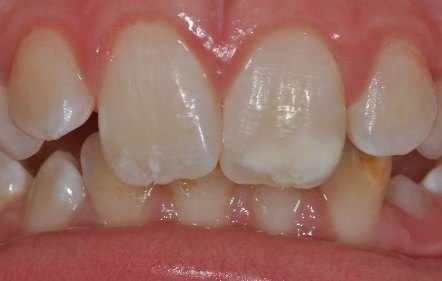 |
| ***F. FOLLOW-UP RELATED QUESTIONS*** |  |  |  |
| 34. | How often do you see these patients post-treatment? | 1. Every 3 months 2. Every 6 months 3. Every 12 months 4. Whenever necessary |  |
| 35. | What are the main clinical findings you see on the recall appointments? | 1. Hypersensitivity 2. Secondary caries 3. Failed restorations 4. Tooth structure loss |  |
| ***G. ATTITUDES AND FUTURE PROPOSALS*** |  |  |  |
| 36. | In which field would you like to improve your knowledge on MIH | 1. Diagnosis 2. Classification 3. Aetiopathogenesis 4. Treatment 5. Follow’up |  |
| 37. | In which way would you like to improve your knowledge on MIH? | 1. Continuous education seminars 2. Webinars 3. Hands-on seminars 4. Study clubs 5. Master Program |  |
